# Real-time thermoregulatory and cardiovascular monitoring of non-acclimatised mountaineers in extreme cold: a 10-day field expedition study

**DOI:** 10.3389/fphys.2026.1727132

**Published:** 2026-03-02

**Authors:** Borja Muniz-Pardos, Panagiotis Verdoukas, Elena Comadran de Barnola, Yiu Chung Issac Chan-Twist, Hashel Al Tunaiji, Yannis Pitsiladis

**Affiliations:** 1 EXER-GENUD (Growth, Exercise, Nutrition and Development) Research Group (S72_23R), FIMS Collaborating Center of Sports Medicine, University of Zaragoza, Zaragoza, Spain; 2 Human Telemetrics LTD, London, United Kingdom; 3 Centre for Exercise Science and Medicine (CESAME), Hong Kong Baptist University, Hong Kong, Hong Kong SAR, China; 4 Zayed Military University, Abu Dhabi, United Arab Emirates; 5 Zayed Military Hospital, Abu Dhabi, United Arab Emirates; 6 UAE National Olympic Committee (NOC) Sport Medicine Committee, Dubai, United Arab Emirates; 7 Department of Biology, Faculty of Science, Hong Kong Baptist University, Hong Kong, Hong Kong SAR, China

**Keywords:** cold stress, core temperature, environmental physiology, heart rate, mountaineering, real-time monitoring, thermoregulation, wearable technology

## Abstract

**Background:**

The primary aim of this study was to characterise the thermoregulatory and cardiovascular responses of non-acclimatised mountaineers during different exercise modalities and camping conditions in extreme cold. A secondary aim was to assess the feasibility of real-time transmission of physiological data to enhance safety during cold expeditions.

**Methods:**

This study assessed thermoregulatory and cardiovascular responses of 18 non-acclimatised mountaineers from the United Arab Emirates during a 10-day winter expedition in Skeikampen, Norway. Participants performed daily cross-country skiing or snowshoe walking (∼5–6 h/day) and experienced two camping conditions (*quinzhee* and tent). Core temperature (Tc) was measured via ingestible telemetric pills, and heart rate (HR) via chest straps. Data were transmitted in real-time using a wearable ecosystem comprising Bluetooth gateways and eSIM-enabled smartwatches.

**Results:**

Cross-country skiing elicited significantly higher mean Tc (+0.20 °C, *p* < 0.01) and HR (+12.8 bpm, *p* < 0.01) than snowshoe walking. Peak Tc during quinzhee camping was significantly higher than during tent camping (+0.55 °C, *p* = 0.03), suggesting superior insulation. No cases of hypothermia were recorded. Real-time data transmission enabled continuous remote monitoring, with actionable alerts triggered when Tc dropped below safety thresholds.

**Conclusion:**

This study demonstrates the feasibility of real-time physiological monitoring in extreme cold, offering practical insight into activity-specific thermoregulatory strain. The findings underscore the importance of shelter design, physical activity selection, and wearable monitoring to enhance safety and decision-making in cold environments.

## 1 Introduction

Athletes and physically active individuals are often at high risk of heat- and cold-related illnesses during training, competition, and outdoor activities (e.g., open water swimming in cold waters, long-distance running, triathlon, or mountain expeditions), which may severely compromise health and performance. Maintaining stable body temperature in the face of such challenging environmental conditions is fundamental for performance and health (Procter et al., 2018). In the cold, the initial response of the body to maintain normal core temperature (Tc) values (approximately 37 °C) includes cold-induced thermogenesis (Schafer et al., 2024). This is defined as an increased energy expenditure above the basal metabolic rate to balance the heat loss to the cold environment and it may involve shivering or nonshivering (Schafer et al., 2024). Cold-induced shivering thermogenesis is associated with involuntary, asynchronous contractions of skeletal muscle to produce heat in an attempt to maintain body temperature with little external work (Arnold et al., 2020). On the other hand, cold-induced nonshivering thermogenesis results from activating brown adipose tissue (van Marken Lichtenbelt and Schrauwen, 2011) and skeletal muscle through protein leak (Blondin et al., 2017), calcium cycling (Ikeda and Yamada, 2022) and other potential mechanisms. Accidental hypothermia refers to an involuntary drop in Tc below 35 °C and is considered a potentially fatal condition, particularly in the presence of trauma (Brown et al., 2012). Of note, the risk of cardiac arrest increases as the Tc drops below 32 °C (Brown et al., 2012). Although severe hypothermia in the absence of trauma is relatively uncommon, mountain environments are particularly hazardous due to combinations of cold, wind, altitude, and remoteness.

Repeated exposure to cold environments induces a process of cold acclimation characterised by physiological adjustments that aim to preserve thermal balance while minimizing energetic cost. These adaptations may include attenuated shivering responses, enhanced non-shivering thermogenesis, altered peripheral vasoconstriction, and improved thermal comfort, although the expression of these responses depends on the intensity, duration, and pattern of cold exposure as well as concurrent physical activity (Castellani and Young, 2016; Daanen and Van Marken Lichtenbelt, 2016).

Field studies in polar and Arctic expeditions suggest that prolonged cold exposure combined with sustained exercise promotes endocrine and metabolic adaptations consistent with cold acclimation, including altered thyroid hormone profiles, activation of thermogenic pathways, and improved tolerance to cold stress (Coker et al., 2017; Gifford et al., 2025; Thuany et al., 2025). In real-world settings, these thermoregulatory challenges are often exacerbated by prolonged exposure, limited recovery opportunities, and logistical constraints inherent to field-based activities in extreme cold environments.

The popularity of recreational activities in the mountains worldwide has led to a growing number of practitioners exposed to extreme environments, increasing the use of helicopter emergency services in some regions (Procter et al., 2018). Off-piste and backcountry winter activities such as ski mountaineering and snowshoe walking have also become increasingly popular, exposing such practitioners to cold ambient temperatures and altitude. In addition to the increased recreational activities in the mountains, military operations are known to include expeditions in extreme environments, testing the limits of human survival (Kingma et al., 2023). Schafer and colleagues (Schafer et al., 2024) recently described the energy expenditure of military service members in cold environments, focusing on the different factors affecting their thermoregulation and causing an excessive energy expenditure during their operational activities. Winter apparel, cold air respiration, inclement weather (e.g., snow, ice) and the type of physical activity directly impact both the energy expenditure and the thermoregulation of the military personnel. Additionally, the combination of cold with altitude, wind and wet environments has been shown to exacerbate overall metabolic strain (Schafer et al., 2024). This increased energy expenditure has been associated with a negative energy balance (exacerbated by the traditional under-eating during military operations due to short breaks, poor appetite and poor palatability of most rations (Marriott, 1995)), dehydration (intensified by limited access to water) and the impairment of physical and cognitive performance (Schafer et al., 2024). As a countermeasure to these challenges, Schafer and colleagues stress the need to introduce wearable technologies into military/mountaineering operations in extreme temperatures for real-time work/rest guidance and early detection of injuries related to cold weather (Schafer et al., 2024).

Field-based studies conducted during cold and polar expeditions have provided important insights into the physiological and metabolic demands of prolonged activity in extreme cold environments. Observational studies and case reports have tracked variables such as energy intake, body mass and composition, hydration status, endocrine responses, substrate utilization, and mood states during Antarctic and Arctic traverses, consistently demonstrating substantial negative energy balance and losses of body mass and lean tissue despite high caloric intake (Paulin et al., 2015; Coker et al., 2017; Thuany et al., 2025).

However, most studies rely on pre- and post-expedition assessments and lack continuous *in situ* measures of thermoregulatory strain, limiting our understanding of how internal temperature is regulated during real-world cold exposure. Real-time monitoring of Tc may therefore provide novel insights into the dynamic interaction between activity, clothing, environment, and metabolic responses during cold expeditions.

Given the above and to complement the existing research, we hypothesised that 1) cross-country skiing would elicit higher Tc and heart rates than snowshoe walking due to its greater aerobic and muscular demands; 2) quinzhee camping would better preserve Tc than tent camping due to superior insulation; and 3) real-time monitoring would allow prompt communication with team leaders in case of any adverse event. For this, the present study aimed to characterise the thermoregulatory and cardiovascular response of non-acclimatised individuals (United Arab Emirates residents) during a 10-day expedition in an extreme cold environment in Skeikampen (Norway). A secondary aim of this research was to transmit the mountaineers’ thermal and cardiovascular data in real time to a mission control room so that the research team could follow their metrics and communicate with the expedition team leader in case of any danger.

## 2 Methods

### 2.1 Study population

A total of 18 mountaineers were recruited for the present study. Thirteen of them were members from the United Arab Emirates (UAE) Mountaineering Expedition Team while the other five were experienced mountaineering instructors (Descriptive characteristics shown in Table 1). Inclusion criteria required participants to not suffer any medical issues prior to the expedition. The study protocol complied with the Declaration of Helsinki and was approved by the local Research Ethics Committee (REC/23–24/0028). This study was part of a series of studies conducted to test the reliability and practicality of use of real-time biometric technology to protect the health of athletes competing in extreme environments (See previous work illustrating the use of this real-time ecosystem (Muniz-Pardos et al., 2019; James et al., 2025)).

**TABLE 1:** Descriptive characteristics of the mountaineers. Mean ± SD; median (interquartile range).

Subject characteristics	All n = 18	UAE mountaineers n = 13	Instructors n = 5
Age (years)	41 ± 10	37 ± 8	51 ± 9*
Height (cm)	174 ± 6	174 ± 7	174 ± 6
Weight (kg)	78.2 ± 12.8	76.9 ± 11.3	81.6 ± 17.0
BMI	25.8 ± 3.1	25.4 ± 2.7	26.6 ± 4.1
V̇O_2_max (mL/kg/min) (n = 10)	-	48.1 ± 4.9	-
Experience in mountaineering (years)	7 (0.5–45)	3 (0.5–12)	26 ± 18*
Number of expeditions in cold environments	3.5 (1–30)	3 (1–25)	23 ± 6*

* Significantly different from UAE, mountaineers.

BMI: body mass index; VO_2_max: maximal aerobic capacity.

All participants wore standardised multi-layer winter clothing selected by the expedition leaders, including a thermal base layer, insulating mid-layers, and a windproof and waterproof outer shell. Clothing was appropriate for Arctic conditions and consistent across participants. This ensured baseline consistency for thermal insulation across activities.

### 2.2 Experimental design

All individuals travelled to Skeikampen (Norway; 800–1 000 m. a.s.l.) during a period of 14 days (6th – 20 February 2024) with the aim to develop their physical and mental fitness in an extreme cold environment. Descriptive data of participants are included in Table 1. Two weeks before the travel to Norway, novice UAE mountaineers only (n = 10; i.e., new members to the UAE expedition team) underwent a maximal oxygen uptake (V̇O_2_max; Cosmed K5, Cosmed Srl, Rome, Italy) test in Sports Medicine Unit (Abu Dhabi, UAE). This test consisted of a walking test to exhaustion with a 10 kg bag on a motorised treadmill (h/p/cosmos, Nußdorf, Germany) at 5.3 km/h and included a 10-min warm-up at 5.3 km/h without extra load followed by 3 min stages with a 10 kg bag and 3% incline increments every 3 min until volitional exhaustion.

**FIGURE 1:**
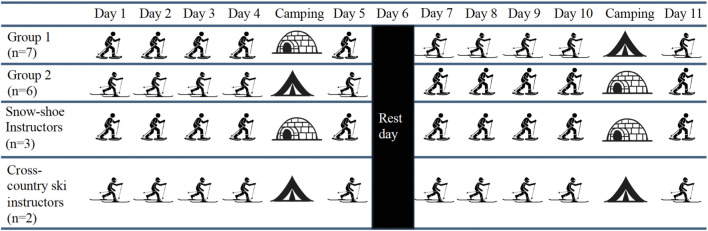
Organization of the exercise and camping activities throughout the expedition for mountaineers and instructors.

**FIGURE 2:**
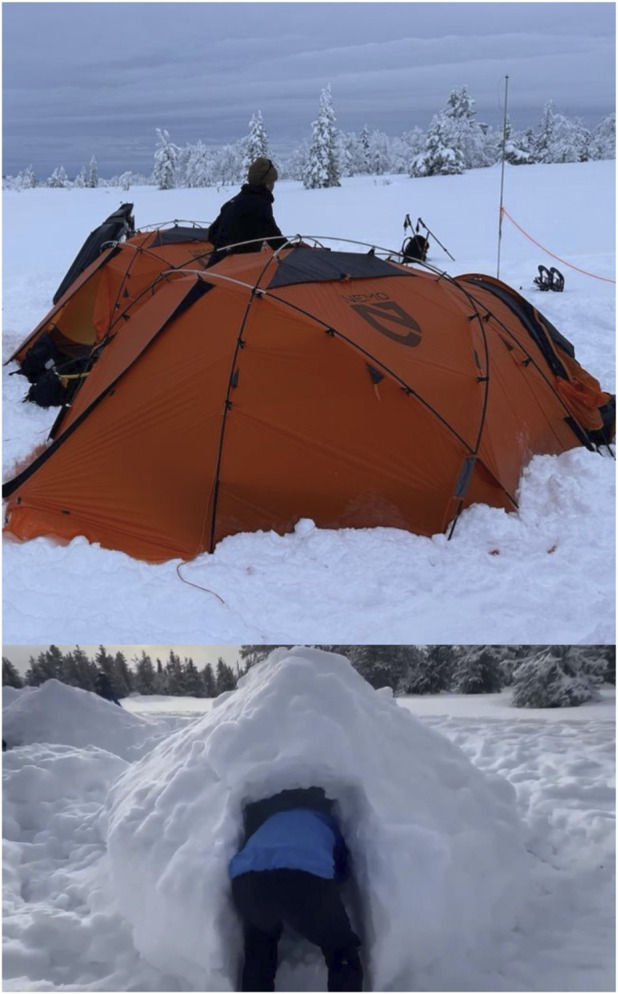
Camping conditions evaluated during the expedition: tent camping (top), *quinzhee* camping (bottom).

The training period consisted of two phases of 5 consecutive days of training with one rest day in between (Figure 1). For the 13 UAE mountaineers, the training regime included 5 days of cross-country skiing and 5 days of snowshoe walking. For this, mountaineers were divided into 2 groups of 7 and 6, and each group began with one of the two activities, and after the resting day, they swapped activities (Table 2). Of note, the 5 instructors remained in the same activity for the 10 days of expedition (two in the ski group and three in the snowshoe walking group). The fourth day of the snowshoe walking activity required mountaineers to camp overnight. Mountaineers involved in snowshoe walking built a *quinzhee* themselves (i.e., a Canadian snow shelter made from a large pile of loose snow that is shaped, then hollowed) to spend the night in the mountains and finish the snowshoe walk training the next morning. However, mountaineers involved in cross-country skiing slept in tents rather than in *quinzhees*. During tent camping, participants slept in four-season mountaineering tents (NEMO Equipment Tenshi™ 2P four-season mountaineering tents) designed for winter use (double-wall construction with an inner breathable fabric and an outer waterproof and windproof polyester shell). Tents were equipped with insulated sleeping mats and winter-rated sleeping bags provided as part of the standard expedition equipment. Figure 2 reflects both camping modalities. Of note, participants spent the same amount of time inside *quinzhees* and tents: 20:00h-06:00h, and the occupancy was of three mountaineers per *quinzhee*/tent.

**TABLE 2:** Day-by-day training program for both activity groups.

Snowshoe walking		
Day	6:30 to 07:00	09:00 to 15:30
1	Morning run (Z1)	Snowshoe – Introduction Snowshoe tour Volume: 8.5 km
2	Morning run (Z1)	Snowshoe – continuation Intro to winter navigation (bearings, pacing with snowshoes) Group management Packing and use of pulks (20–25 kg) Tour with pulks (20–25 kg) Volume: 8.9 km
3	Morning run (Z1)	Winter skills 1. Use of ice axe 2. Step cutting, self-belay, ice axe belays 3. Self-arrest 4. Emergency shelters Volume: 7.7 km
4	Preparation for expedition	Snowshoe tour and camping Volume: 7.4 km
5	Expedition	Volume: 5.1 km

Cross-country skiing		
Day	6:30 to 07:00	09:00 to 15:30
1	Morning run (Z1)	9:00 to 12:00: Technical drills 13:30 to 15:30 Volume: 7 km
2	Morning run (Z1)	9:00 to 12:00: Technical drills 13:30 to 15:30: 10 km ski tour Volume: 14 km
3	Morning run (Z1)	9:00 to 12:00: Technical drills 13:30 to 15:30: 10 km ski tour Volume: 19 km
4	Morning run (Z1)	Cross-country tour and camping 9:00 to 12:00: Technical drills 13:30 to 15:30: 10 km ski tour Volume: 20 km
5	-	9:00 to 15:30 Volume: 22 km

Forcross-country skiing, technical drills included: classic and skating technique practice, glide control, hill climbing/descending, balance drills, pole plant timing and pole-leg synchronization, braking, turning, pacing.

All mountaineers performed a low-intensity run at 0630 during 30 min (Z1) and thereafter ingested a telemetric pill (e-CelsiusTM, BodyCap Inc, France) at 0700, had breakfast, prepared the equipment, and started their corresponding activity from 0900 to 1530. A detailed explanation of the exercise performed each day is shown in Table 2. Participants consumed a light lunch between approximately 12:00 and 13:30, depending on expedition logistics and environmental conditions. Meals consisted of typical portable foods used during cold-environment mountaineering (e.g., energy bars, dried foods, soup). Energy intake was not quantitatively assessed, as dietary intake was not standardized and continuous monitoring of food consumption was not feasible under the field conditions of the expedition. No large meals were consumed during the experimental period. Fluid intake (water or warm beverages) was allowed *ad libitum* throughout the expedition.

Telemetric pills have shown excellent utility to test Tc in field studies such as distance running or also during sustained military training exercises in soldiers (Byrne and Lim, 2007). The ingestion of these telemetric capsules has shown excellent tolerability, having no collateral issues. Individuals unable to ingest it orally, are usually recommended to insert it rectally. However, this was not necessary for this study. Capsules are naturally excreted and not retrieved, which is standard practice in human field studies with ingestible temperature pills. Each mountaineer was required to wear a heart rate belt (Polar H9, Polar Electro, Kempele, Finland), carry a gateway (BodyCap Inc, France; receptor of the radio-wave emitted by the pill at a frequency of 60 Hz) in a pocket, close to the gastrointestinal tract, and wear a smartwatch (Samsung Pro 5, Samsung, Seoul, South Korea) equipped with an electronic SIM. This was connected via Bluetooth to the gateway, so that their Tc, heart rate and geolocation data could be transmitted in real time to the technical team located in a mission control room in a hotel (for further details explaining the functioning of this ecosystem of real-time monitoring, see previous research (Muniz-Pardos et al., 2019)). Of note, while these devices have been validated in laboratory and field conditions, extreme cold may affect battery performance and signal quality. The present research was used as a feasibility study for the use of such technology in extreme cold conditions. Smart watches were attached to portable batteries (Svartgoti, CYT02, 10,000 mAh/37 Wh) so these were constantly charging. During camping, an extra battery was provided to mountaineers to allow for sufficient battery. Environmental conditions were registered using a Kestrel 5400 heat stress tracker (Nielsen-Kellerman Co., Boothwyn, PA, USA; temperature range = −29.0 °C–70.0 °C) in the nearby area of the starting point each day. Values for ambient temperature, humidity, heat index, wind speed and wet bulb globe temperature (WBGT) were collected.

Participants wore a standardized multi-layer cold-weather clothing system appropriate for Arctic conditions. This system consisted of a moisture-wicking base layer (top and bottom), one or two insulating mid-layers (fleece or synthetic insulation), and a windproof and water-resistant outer shell. Insulated gloves, cold-weather boots, and head insulation (hat or balaclava) were worn throughout the expedition. Intrinsic clothing insulation (clo), clothing area factor, and evaporative resistance were not measured, as these require laboratory-based garment testing and could not be obtained under field conditions. Clothing mass was not recorded.

In order to guarantee complete Tc data during camping (mountaineers left the base camp in the morning and stayed out until 30 h later), a first telemetric pill was ingested in the morning and a second pill in the evening before dinner and camping (at ∼19:30), so that overnight Tc was guaranteed. Given that all participants retained the first pill ingested in the morning during the first camping test (*quinzhee*), mountaineers only ingested the first pill in the morning during the second camping test (tent).

### 2.3 Training program

Detailed tasks during the 10-day training program are displayed in Table 2.

### 2.4 Statistical analysis

Descriptive statistics were computed for mean, minimum and maximum Tc and heart rate across activities and days. Assumptions of normality were checked through the Shapiro-Wilk test. When normality assumptions were violated, non-parametric alternatives were applied. Differences in mean, minimum, and maximum Tc and heart rate across time and exercise activities were analysed using linear mixed-effects models (LMMs), with Time (days 1–10) and Activity (cross-country skiing, snowshoe walking) included as fixed effects, along with their interaction (Time × Activity). Subject was included as a random effect to account for repeated measures. When significant interaction was observed, *post hoc* pairwise comparisons were adjusted using the Bonferroni correction to control for multiple testing. Adjusted *p*-values were compared against a significance threshold of α = 0.05. Differences in mean, minimum and maximum Tc/heart rate between camping activities (*quinzhee*, tent) were further explored through one-way ANOVA, as these comparisons involved independent camping conditions. Differences between mean, minimum and maximum Tc between the two pills ingested during *quinzhee* camping were examined through Student’s *t*-tests. We performed all analyses using JAMOVI statistical software. Figures were generated using Labplot2 software. Statistical significance was set at *p* < 0.05.

## 3 Results

Expedition instructors showed a significantly higher age (χ^2^ = 5.4, ε^2^ = 0.32, *p* = 0.02), years of experience (χ^2^ = 5.1, ε^2^ = 0.30, *p* = 0.03) and number of expeditions (χ^2^ = 5.5, ε^2^ = 0.32, *p* = 0.02; Table 1), when compared to mountaineers. From the 18 mountaineers over the 10-day training period, 10 completed all days of training. Five missed 1 day due to the following reasons: arriving 1 day later (n = 1), respiratory infection (n = 1), knee injury (n = 1), Achilles tendinitis (n = 1), and personal issues (n = 1). One mountaineer missed 2 days due to hip flexor muscle injury (n = 1), another missed 3 days due to knee injury (n = 1), and the 18th mountaineer only participated in the 5 days ski activity, missing the 5 snowshoe training days (n = 1). The day-to-day participation over the whole expedition is shown in Table 3.

**TABLE 3:** Day-to-day compliance of the mountaineers during the 10-day expedition.

Group	Day 1	Day 2	Day 3	Day 4	Camping	Day 5	Day 6	Day 7	Day 8	Day 9	Camping	Day 10
Ski group	8	9	9	7		7	9	9	9	9		7
Snowshoe group	9	9	9	9	8	10	7	8	7	7	7	7
Total	17	18	18	16	8	17	16	17	16	16	7	14

### 3.1 Environmental conditions

The data collected by the Kestrel device during the expedition are shown in Table 4. One-way ANOVA revealed a significantly colder temperature during the first week, when compared to the second week in ambient temperature (*p* = 0.01; F = 10.9; Table 4), WBGT (*p* = 0.04; F = 7.9; Table 4) and Heat index (*p* = 0.02; F = 11.1; Table 4). The route profiles during the expedition included rolling terrain with elevations (800–1 000 m. a.s.l.) including slopes and open sections, with limited tree cover. This was accompanied by frequent strong winds in open sections as perceived by mountaineers. Very firm, fast snow was found on exposed areas, while this was soft powder in leeward zones.

**TABLE 4:** Environmental data. Mean ± SD.

Time period	Ambient temperature (^o^C)	Humidity (%)	Wind speed (km/h)	WBGT (^o^C)	Heat index
All days	−8.5 ± 6.3	79 ± 17	0.7 ± 1.6	−6.8 ± 6.9	−7.1 ± 6.5
First week	−13.1 ± 4.0*	84.6 ± 11.6	1.1 ± 3.2	−11.5 ± 5.8*	−12.2 ± 3.5*
Second week	−4.0 ± 4.8	74.4 ± 20.1	1.3 ± 2.1	−3.1 ± 5.6	−3.1 ± 5.4

WBGT , wet bulb globe temperature.

* Significantly different to Second week (*p*< 0.05).

### 3.2 Thermoregulatory response

**FIGURE 3:**
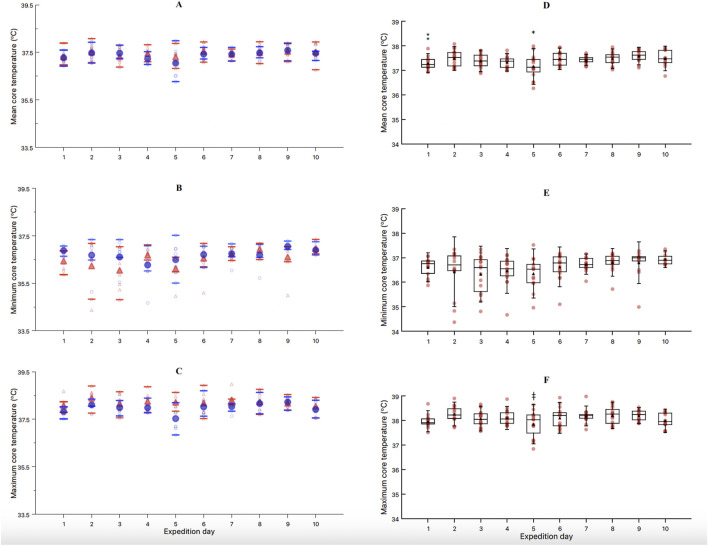
Mean **(A)**, minimum **(B)** and maximum **(C)** core temperature between showshoe walking (blue circles) and cross-country skiing (red triangles) across the 10 days of expedition. Mean **(D)**, minimum **(E)** and maximum **(F)** core temperature through the 10-day expedition, regardless of the exercise activity. *Significantly different mean core temperature than days 2,6,7,8,9 and 10. ⁑significantly different mean core temperature than day 9. ǂsignificantly different than days 2 and 8. Individual data, mean and SD are presented for each exercise activity and expedition day.

**FIGURE 4:**
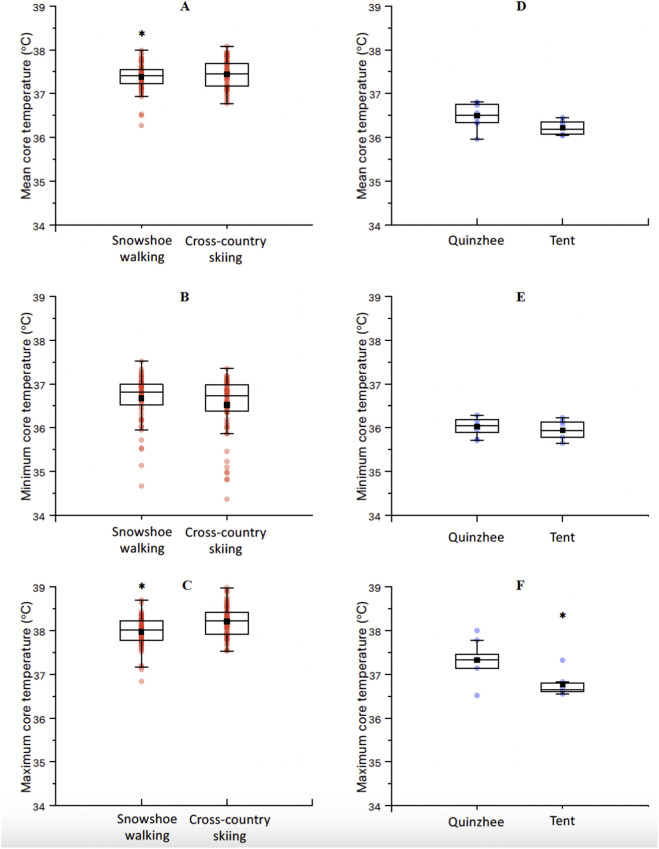
Mean **(A)**, minimum **(B)** and maximum **(C)** core temperature between the two exercise activities, regardless of the day of the expedition. Mean **(D)**, minimum **(E)** and maximum **(F)** core temperature between the two camping activities (*quinzhee* [n = 7] and tent [n = 6]). *Significantly different.

For the mean Tc, the results from the LMM revealed a significant effect of TIME (F = 4.46, *p* < 0.01) and ACTIVITY (F = 25.76, *p* < 0.01). The interaction DAY*ACTIVITY showed a non-significant trend (F = 1.89, *p* = 0.06, Figures 3A–C). The *post hoc* analysis with Bonferroni correction for the factor TIME (Figures 3D–F) showed significant differences between day 1 and 9 (−0.27 °C, t = −3.5, *p* = 0.03), day 2 and 5 (0.33 °C, t = 4.6, *p* < 0.01), day 5 and 6 (−0.28 °C, t = −3.8, *p* = 0.01), day 5 and 7 (−0.25 °C, t = −3.4, *p* = 0.04), day 5 and 8 (−0.29 °C, t = −4.0, *p* = 0.01), day 5 and 9 (−0.40 °C, t = −5.4, *p* < 0.01), and day 5 and 10 (−0.26 °C, t = −3.4, *p* = 0.05). Additionally, *post hoc* analysis with Bonferroni correction for the factor ACTIVITY showed significant differences between cross-country ski and snowshoe walking (0.20 °C, t = 5.0, *p* < 0.01, Figures 4A–C).

For the minimum Tc, the LMM showed no significant effect of TIME (Figure 3E), ACTIVITY (Figure 4B), nor their interaction (Figure 3B). Finally, for the maximum Tc, the LMM showed a significant effect of TIME (F = 56.13, *p* < 0.01, Figure 3F) and ACTIVITY (F = 25.76, *p* < 0.01, Figure 4C). The interaction DAY*ACTIVITY showed a non-significant trend (F = 1.89, *p* = 0.06, Figure 3C). The *post hoc* analysis for TIME showed significant differences between day 2 and 5 (−0.37 °C, t = 4.0, *p* = 0.01), and between day 5 and 8 (−0.31 °C, t = 3.35, *p* < 0.05). The *post hoc* analysis for the maximum temperature according to the factor ACTIVITY showed significant differences between cross-country ski and snowshoe walking (0.36 °C, t = 6.90, *p* < 0.01, Figure 4C).

Finally, the one-way ANOVA showed that mean (F = 4.31, *p* = 0.06) and minimum (F = 0.44, *p* = 0.52) Tc were not different between camping activities. However, the maximum Tc during the tent camping showed to be lower when compared to quinzhee camping (−0.55 °C, F = 6.01, t = 2.45, *p* = 0.03, Figure 4F).

As described in the methods section, during the quinzhee camping each mountaineer ingested two pills (the first pill at 07:00 and a second pill before dinner, at ∼19:30). Data from both pills were obtained from six mountaineers. Paired sample t-test showed no differences in the mean (mean difference = 0.05 °C, t = 0.98, *p* = 0.37), minimum (mean difference = 0.13 °C, t = 1.53, *p* = 0.19), and maximum (mean difference = 0.05 °C, t = 0.87, *p* = 0.44) Tc recorded by both pills during camping.

In an additional exploratory analysis, ambient temperature was introduced as a covariate in a linear mixed-effects model to predict mean Tc. This model was not significant (r^2^ = 0.031; *p* = 0.10), illustrating that colder days were not associated with lower mean Tc after accounting for activity.

### 3.3 Cardiovascular response

**FIGURE 5:**
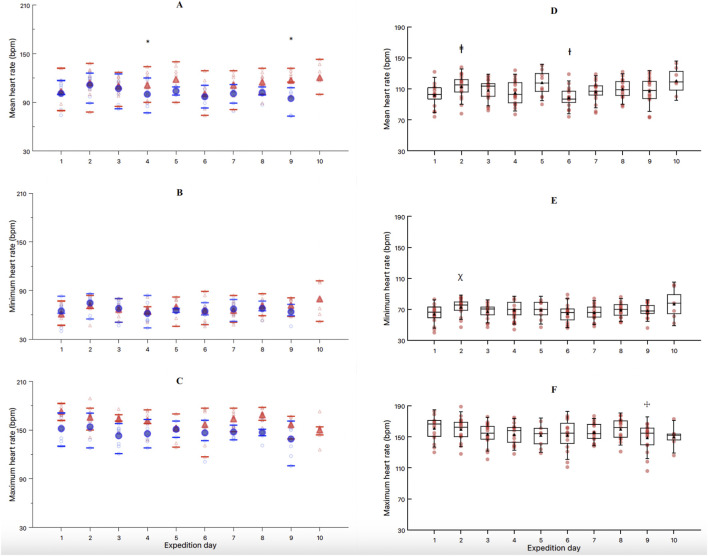
Mean **(A)**, minimum **(B)** and maximum **(C)** heart rate between showshoe walking (blue circles) and cross-country skiing (red triangles) across the 10 days of expedition. Mean **(D)**, minimum **(E)** and maximum **(F)** heart rate across the 10 days of expedition, irrespective of exercise activity. * = significantly different between activities; ⱡ significantly different to day 1; ᵻ significantly different to days 2, 3 and 4; ꭓ significantly different to days 1 and 4. ☩significantly different to day 1 and 2. Individual data, mean and SD are presented for each exercise activity and expedition day.

**FIGURE 6:**
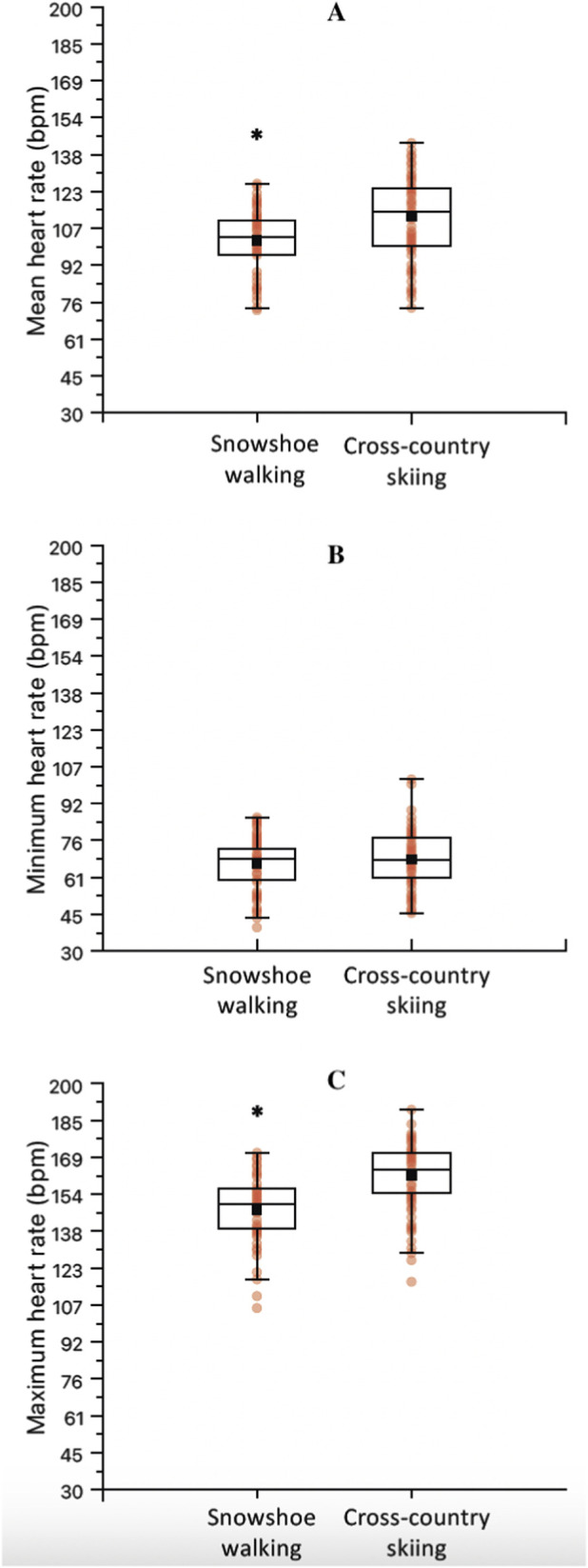
Mean **(A)**, minimum **(B)** and maximum **(C)** heart rate between the two exercise activities, irrespective of the day of the expedition. *Significantly different.

For the mean heart rate, the results from the LMM revealed a significant effect TIME (F = 5.50, *p* < 0.01, Figure 5D), ACTIVITY (F = 66.06, *p* < 0.01, Figure 6A) and TIME*ACTIVITY interaction (F = 3.43, *p* = 0.01, Figure 5A). Further *post hoc* analysis revealed a significantly reduced mean heart rate during snowshoe walking when compared to cross-country skiing, overtime, during day 4 (−18.5 bpm, t = 4.87, *p* < 0.01) and day 9 (−22.3 bpm, t = 5.93, *p* < 0.01) (Figure 5A). Additionally, *post hoc* analysis of TIME, regardless of the ACTIVITY performed, showed a significantly greater mean heart rate during day 2 when compared to day 1 (−9.0 bpm, t = −3.82, *p* = 0.01), and a reduced mean heart rate during day 6 when compared to days 2 (−13.0 bpm, t = 5.55, *p* < 0.01), 3 (−8.7 bpm, t = 3.70, *p* = 0.01), and 4 (−8.0 bpm, t = 3.30, p < 0.05) (Figure 5D). Finally, the *post hoc* analysis of the mean heart rate between the two activities regardless of TIME, showed a reduced mean heart rate during snowshoe walking when compared to cross-country skiing (−12.8 bpm, t = 7.70, *p* < 0.01, Figure 6A).

For minimum heart rate, there was no significant effect of ACTIVITY (F = 3.17, *p* = 0.08, Figure 6B) nor TIME*ACTIVITY interaction (F = 1.68, *p* = 0.11, Figure 5B). However, there was a significant effect of TIME (F = 4.63, *p* < 0.01, Figure 5E). Post-hoc pairwise comparisons for TIME revealed a significantly higher minimum heart rate during the day 2, when compared to day 1 (7.8 bpm, t = −3.51, *p* = 0.02) and day 4 (8.6 bpm, t = 3.95, *p* = 0.01) (Figure 5E).

Finally, for maximum heart rate, there was a significant effect of TIME (F = 4.48, *p* < 0.01, Figure 5F) and ACTIVITY (F = 45.92, *p* < 0.01, Figure 6C), but no significant TIME*ACTIVITY interaction (F = 1.24, *p* = 0.28, Figure 5C). Bonferroni *post hoc* pairwise comparisons for TIME showed a significantly lower maximum heart rate during day 9, when compared to days 1 (15.2 bpm, t = 4.27, *p* = 0.01) and 2 (12.0 bpm, t = 3.55, *p* = 0.02) (Figure 5F). For ACTIVITY, Bonferroni *post hoc* pairwise comparisons showed a significantly reduced maximum heart rate for snowshoe walking when compared to cross-country skiing (14.3 bpm, t = 6.24, *p* < 0.01, Figure 6C).

### 3.4 Operational considerations

One of the main obstacles faced before the expedition was the battery performance in sub-zero conditions and the cellular connectivity to transmit in real time in remote areas. Our set up with the smart watches attached to external batteries allowed for optimal battery performance throughout the expedition, with mountaineers coming back with 100% battery in their smart watch. Overall, we could follow the data in real time from the mission control room. We obtained intermittent cellular coverage in some cases but the mobile app was designed to retrieve and upload all data automatically once signal returned, which was successfully performed. Given that cellular connectivity was reasonably stable throughout the expedition, we could contact the team leader in case Tc of any of the mountaineers approached 35 °C. This happened in one occasion and hot drink and extra cover was immediately provided to such affected individual and no severe symptoms occurred.

## 4 Discussion

To our knowledge, the present research is the first to report the thermoregulatory and cardiovascular responses of mountaineers from a warm origin (UAE residents) during a 10-day expedition in a cold environment (Skeikampen, Norway). Participants engaged in extensive exercise (∼5–6 h/day) and were exposed to two different exercise modalities (cross-country skiing and snowshoe walking) alongside two camping modalities (tent and *quinzhee* camping).

During the expedition, no cases of hypothermia were reported, despite the lowest Tc values being recorded during camping, ranging from 35.7 °C to 36.3 °C during quinzhee camping and from 35.6 °C to 36.2 °C during tent camping. Peak Tc, however, were significantly greater during quinzhee camping than during tent camping. Notably, environmental conditions were significantly colder during week 1, when compared to week 2 (Table 4). This could influence the Tc of mountaineers during the first week, although the cross-over design of this experiment with both exercise and camping activities aimed to prevent from this bias. Importantly, an exploratory mixed-effects model including ambient temperature as a covariate showed no significant association with mean Tc, suggesting that activity type rather than environmental variation primarily drove thermoregulatory responses. While our results should be interpreted with caution because we did not measure ambient temperatures within tents/quinzhees, the greater Tc observed in quinzhees aligns with Alford et al., who highlighted the advantages of snowhouses over tents, suggesting that snow structures provide a warmer environment, especially during freezing temperatures (Alford, 1999). Zhen et al. also supported this, noting that igloos create a comfortable thermal environment, being approximately 4 °C warmer inside than outside in extremely cold settings (Zhen et al., 2021). The greater peak Tc observed in mountaineers camping in quinzhees compared to those in tents may be attributed to these thermal benefits. Additionally, mountaineers camping in *quinzhees* built the snow shelters themselves, which could have potentially impacted their pre-sleep fatigue and metabolic heat production given such strenuous activity when compared to tents set up. This could have explained the significantly greater peak Tc observed during quinzhee camping (Figure 4F).

Notably, prior to quinzhee camping on day 4 of the expedition, participants ingested a second telemetric pill in the evening to ensure accurate Tc readings. The absence of significant differences in Tc readings between the two pills may be due to sufficient time for digestion during the night and limited fluid intake while sleeping (Byrne and Lim, 2007).

The environmental conditions during the first 5 days of the expedition were more challenging than those during the second half, which may explain the reduced mean (−0.1 °C) and minimum (−0.3 °C) Tc during the first week. Although no significant differences were observed in Tc or heart rates between exercise activities across the 10-day period, cross-country skiing elicited higher peak Tc, accompanied by increased mean and peak heart rates compared to snowshoe walking. This is likely due to the greater energy demand and muscle mass involvement in cross-country skiing, as demonstrated by previous studies that identified peak aerobic power as a key performance indicator for this sport (Norman and Komi, 1987; Losnegard and Hallén, 2014). Snowshoe walking, by contrast, is associated with lower energy requirements, emphasizing the need for careful consideration of energy expenditure in relation to thermal strain and other risks, such as dehydration and fatigue (Connolly, 2002). A great limitation of our research is that we did not measure energy expenditure, which restricts our ability to quantify the relationship between cold-induced thermogenesis, physical work, and Tc responses.

Mountaineers and instructors travelled for ∼12 h from the UAE to Norway (i.e., East to West), with a difference of three time-zones. While jet-lag symptoms have shown to worsen when traveling East, compared to West (Thun et al., 2015), research on jet-lag recovery timeline is limited. In this regard, a previous study in professional athletes reported increased subjective jet-lag ratings for 5 days following a long-haul travel across 1 time zone (Fowler et al., 2015). Of note, this was suggested to be explained due to sleep disruption induced by an early departure time rather than circadian rhythm misalignment. The long trip performed in the present study together with the 3 h time-zone difference may have impacted mountaineers’ perceived fatigue and, potentially, circadian rhythm during the first days of the expedition. Human thermoregulatory responses are superimposed upon circadian changes in Tc that originate from the suprachiasmatic nuclei at the base of the hypothalamus (Waterhouse et al., 2005). Tc has shown to peak during in the late evening, and minimum in the morning, with a resting individual being in a “heat gain” mode in the morning and in a “heat loss” mode in the evening (Waterhouse et al., 2005). In our study, the Tc of an individual potentially suffering from jet-lag symptoms during the first days would have likely reflected an impaired thermoregulatory response at rest and during exercise. However, our study did not include measurements of sleep quality or jet-lag symptoms.

**FIGURE 7:**
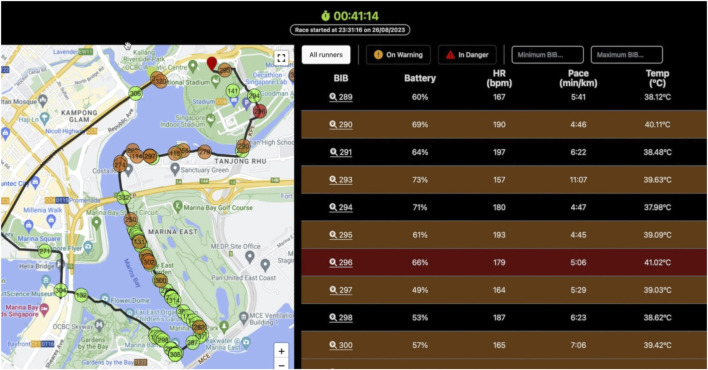
Traffic light system to predict exertional heat stroke during the 2023 SAFRA Singapore Bay Run – 10 .km.

Our study also successfully piloted the real-time transmission of Tc, cardiovascular responses, and geographical location. Data transmission was feasible due to adequate cellular signals in mountainous terrains. During quinzhee camping, we detected one participant whose Tc fell below 35.5 °C, allowing for timely intervention by the expedition team leader, thereby underscoring the importance of continuous monitoring in ensuring participant safety. In the past, our team has implemented this real-time technology in different scenarios, such as the 2022 adidas Road to Records event (Guppy et al., 2023), the 2021 Brighton marathon (Grivas et al., 2024), the 2020 Tokyo Olympics (Guppy et al., 2023), the 2024 Hong-Kong triathlon World Cup (James et al., 2025), a 200-km ultra endurance race across the desert (Esh et al., 2025), and during the 2023 Singapore Bay Run (Killoughery and Pitsiladis, 2024). With this, our team designed a traffic light system solution for the first time to predict risk of exertional heat stroke (See example in Figure 7), which was piloted in Norway for first time in extreme cold conditions.

The implementation of this technology not only in mountaineering but also in other endurance activities conducted in challenging or remote environments (e.g., trail running, marathon and ultra-marathon, open water swimming, cycling) would allow for a prompt intervention in case of any danger. Long-lasting sporting events which include abrupt changes in environmental conditions such as open water swimming (in both cold and hot waters) (Tipton and Bradford, 2014) or trail running (Parise and Hoffman, 2011) which may include highly variable conditions (snow, heavy rain, wind) pose a greater risk for athletes, with an additional risk related to the challenges faced by medical teams to access/evacuate athletes from remote locations. In 2021, a number of athletes participated in a 100-m ultra-marathon in northern China (BBC, 2021). During the race, environmental conditions did not follow the forecast predictions and became extremely dangerous including hail, heavy rain and gales, causing temperatures to plummet. A total of 172 runners went missing and a rescue operation was initiated. Despite all the efforts, 21 runners died and many suffered from hypothermia and other injuries, with Chinese authorities suspending all high-risk sporting events (such as trail running, desert tracking, wingsuit flying and ultra-marathons) that lack oversight, rules and safety standards. This traumatic event illustrates the severe risks that insufficient preparedness, inadequate safety protocols, and delayed medical response can pose during endurance events conducted in cold and remote environments.

The results of this study highlight the importance of structured training in severe cold environments to enhance performance in mountaineering. The combination of cross-country skiing and snowshoe walking in a training regimen can facilitate comprehensive physiological adaptation. This is crucial for athletes preparing for the unique challenges posed by high-altitude expeditions (Kreher and Schwartz, 2012). The differences in physiological responses between exercise activities inform expedition leaders about appropriate countermeasures, such as ensuring sufficient hydration, energy intake, and adequate rest breaks to mitigate risks associated with prolonged physical activity in cold conditions. Field data during Antarctic expeditions consistently demonstrate that daily energy expenditures often exceed 5,000–8,000 kcal·day^-1^, resulting in sustained energy deficits (Gifford et al., 2025; Thuany et al., 2025). An additional consideration is the shift in substrate utilization in cold conditions. Paulin and colleagues reported that athletes completing a 800-km Antarctic ultra-endurance race derived over 60% of their energy intake from fat, with carbohydrate contributing less than 25% (Paulin et al., 2015). This reflects logistical food constraints and gastrointestinal tolerance under extreme cold conditions, which should be considered by expedition team leaders. A recent systematic review on Antarctic expeditions (Thuany et al., 2025) highlights that sustained body mass loss (including fat-free mass) occurs despite high caloric intakes, reflecting a persistent negative energy balance driven by the combined demands of 1) prolonged physical activity, 2) cold-induced thermogenesis, 3) impaired substrate utilization and 4) hypoxic exposure. These conditions elicit coordinated endocrine and metabolic efforts to prioritise survival and optimise work output at the expense of lean tissue preservation (Thuany et al., 2025).

While our study failed to measure energy expenditure, our findings provide complementary, valuable and novel data about the thermoregulatory and cardiovascular response of non-acclimatised individuals to extreme cold over a 10-day period, with unprecedented real-time monitoring in these conditions. Overall, we found that the selection of more demanding activities (e.g., cross-country skiing) would be associated with a reduced drop in Tc and greater fatigue. While these activities would increase metabolic heat produced by the practitioners, it would require greater attention to rest periods, and an increased amount of energy intake to avoid excessive levels of fatigue in such a challenging environment. Our observations during camping highlight the importance of selecting the safest camping modality, especially when cold is more severe. The use of snowhouses would be preferable over tents to ensure an optimised insulation and prevent Tc from dropping to high-risk values. Special attention should be paid to clothing, since this can potentially modify energy expenditure. Adequate insulation may blunt thermoregulatory-driven increases in resting metabolic rate by maintaining thermal comfort, as suggested in modern Antarctic traverses where participants reported minimal cold discomfort and no clear post-expedition elevation in their resting metabolic rate (Hattersley et al., 2025). Finally, the implementation of real-time monitoring of Tc, heart rate and geolocation successfully allowed for early identification of excessively low Tc, permitting an immediate communication with team leaders.

## 5 Limitations

Despite the valuable insights gained from this study, several limitations should be acknowledged. The sample size of 18 participants may limit the generalizability of the findings. Future research should aim for larger sample sizes to validate these results across various populations and skill levels. The inclusion of females within the sample would also reveal potential sex-specific differences. Additionally, the absence of a control group restricts the ability to attribute the observed changes to the training interventions. Incorporating a control group in future studies could enhance the understanding of the effects of training in extreme conditions.

The design of the study did not include measurements of other relevant variables such as skin temperature (relevant for other cold-related risks such as frostbite), energy intake, energy expenditure, or fluid intake during the expedition. This limitation did not allow us to quantify energy balance, hydration status or directly link metabolic cost to thermoregulatory outcomes. While the same winter apparel was provided, the absence of fat-mass and body composition data limits our ability to interpret inter-individual differences in cold tolerance and thermoregulation. Clothing insulation parameters, including intrinsic insulation (clo), clothing area factor, and evaporative resistance, were not quantified, as laboratory-based garment testing was not feasible during the expedition. While the clothing layering system is fully documented, the absence of measured insulation indices prevents detailed heat-balance modelling. Lastly, the environmental conditions fluctuated throughout the expedition, complicating comparisons of thermal stress between different camping modalities. However, the crossover design, where participants changed exercise activities after 5 days, minimised potential bias from these varying conditions. For camping modalities, we did not measure ambient temperatures inside tents/quinzhees but only Tc which limits the insulation capacity of these camping modalities and their comparison.

During our study, real-time monitoring was possible because of a reasonably stable cellular connectivity. Adoption in other remote settings will require robust solutions for areas without cellular signal, potentially via satellite-enabled devices. Future investigations should further explore the physiological and psychological responses of mountaineers over extended periods in extreme conditions. The incorporation of direct energy expenditure measures will be crucial in future studies, given the importance of negative energy balance and under-eating highlighted by Schafer et al. and others (Schafer et al., 2024). Specific areas of interest could include nutritional strategies, recovery protocols, and mental training techniques that enhance performance during prolonged expeditions. Additionally, advanced monitoring technologies, including wearable sensors and machine learning algorithms, could provide deeper insights into real-time physiological changes and individual responses to training stimuli.

## 6 Conclusion

This study provides novel field-based evidence on the thermoregulatory and cardiovascular responses of non-acclimatised individuals during prolonged exposure to extreme cold, using real-time physiological monitoring. Our findings demonstrate that activity selection and shelter design meaningfully influence Tc regulation, with cross-country skiing and quinzhee shelters eliciting higher peak Tc, which suggest potentially greater thermal protection. Importantly, this study shows that continuous real-time monitoring of Tc and heart rate is feasible in Arctic environments and can enable timely interventions to enhance safety. These findings have direct implications for the planning, training, and risk management of mountaineering, military, and endurance activities in extreme cold conditions.

## Data Availability

The original contributions presented in the study are included in the article/supplementary material, further inquiries can be directed to the corresponding author.
